# Association between six-minute walk test parameters and the health-related quality of life in patients with pulmonary *Mycobacterium avium* complex disease

**DOI:** 10.1186/s12890-018-0686-5

**Published:** 2018-07-13

**Authors:** Kazuma Yagi, Takanori Asakura, Ho Namkoong, Shoji Suzuki, Takahiro Asami, Satoshi Okamori, Tatsuya Kusumoto, Yohei Funatsu, Hirofumi Kamata, Tomoyasu Nishimura, Makoto Ishii, Tomoko Betsuyaku, Naoki Hasegawa

**Affiliations:** 1 Division of Pulmonary Medicine, Department of Medicine, Keio University School of Medicine, Tokyo, Japan; 2 Keio University Health Center, Tokyo, Japan; 3 Center for Infectious Diseases and Infection Control, Keio University School of Medicine, 35 Shinanomachi Shinjuku, Tokyo, 160-8582 Japan

**Keywords:** Nontuberculous mycobacteria, *Mycobacterium avium* complex, Six-minute walk test, Health-related quality of life, 36-item short form health survey, St. George’s respiratory questionnaire

## Abstract

**Background:**

Pulmonary *Mycobacterium avium* complex (pMAC) disease is a chronic, slowly progressive disease. The aim of the present study was to determine the association of six-minute walk test (6MWT) parameters with pulmonary function and the health-related quality of life (HRQL) in patients with pMAC disease.

**Methods:**

This cross-sectional study included adult patients with pMAC and was conducted at Keio University Hospital. We investigated the relationship of 6MWT parameters with clinical parameters, including pulmonary function, and HRQL, which was assessed using the 36-Item Short Form Health Survey (SF-36) and St. George’s Respiratory Questionnaire (SGRQ).

**Results:**

In total, 103 consecutive patients with pMAC participated in 6MWT (median age, 64 years; 80 women) and completed SF-36 and SGRQ. The six-minute walk distance (6MWD) showed significant negative and positive correlations with all SGRQ domain scores [ρ = (− 0.54)–(− 0.32)] and the physical component summary (PCS) score (ρ = 0.39) in SF-36, respectively; the opposite was observed for the final Borg scale (FBS) score (all SGRQ scores, ρ = 0.34–0.58; PCS score, ρ = − 0.50). The distance-saturation product showed significant negative and positive correlations with all SGRQ scores [ρ = (− 0.29)–(− 0.55)] and the PCS score (ρ = 0.40), respectively. Multivariate analysis revealed that 6MWD and the FBS score were significant predictors of HRQL.

**Conclusions:**

Our findings suggest that 6MWD and the FBS score are useful parameters for evaluating HRQL in patients with pMAC. Further studies should investigate the impact of 6WMT parameters on disease progression, treatment responses, and prognosis.

**Electronic supplementary material:**

The online version of this article (10.1186/s12890-018-0686-5) contains supplementary material, which is available to authorized users.

## Background

The increasing prevalence of pulmonary infections due to nontuberculous mycobacteria (NTM) is an emerging public health concern worldwide [1, 2]. Pulmonary *Mycobacterium avium* complex (pMAC) disease, the most common form of NTM infection, presents as a chronic, slowly progressive disease in immunocompetent patients [3]. It causes chronic pulmonary diseases such as asthma, chronic obstructive pulmonary disease (COPD), and interstitial lung disease (ILD) and is generally incurable, requires long-term antimicrobial therapy, and has a high recurrence rate after treatment discontinuation [3]. Because of the increasing chronicity of pMAC disease, patient-reported outcome measures that represent the health-related quality of life (HRQL) have become increasingly important to monitor the overall health status of affected patients [4]. It has been reported that HRQL, particularly the physical component, is impaired in patients with pMAC [5].

The six-minute walk test (6MWT) has become a useful tool for assessing the functional status and predicting the prognosis of patients with various pulmonary diseases, including COPD, ILD, sarcoidosis, and primary pulmonary hypertension [6–9]. It is also used as a standardized exercise test for the assessment of lung disease because of its simplicity, low cost, non-invasiveness, ease of performance, and reproducibility [10, 11]. Recently, a randomized study of inhaled liposomal amikacin revealed an improvement in 6MWT parameters in patients with pNTM disease [12]. With regard to the clinical significance of 6MWT for pMAC disease, only one study has assessed the relationship between the six-minute walk distance (6MWD) and HRQL evaluated using St. George’s Respiratory Questionnaire (SGRQ) [13]. However, the correlations between HRQL and various 6MWT parameters remain unknown.

The aim of the present study was to investigate the relationship of 6MWT parameters with clinical parameters, including pulmonary function test (PFT) findings, and HRQL. The tested hypothesis was that 6MWT parameters could be useful for evaluating HRQL in patients with pMAC disease.

## Methods

### Study design and study population

This cross-sectional study included adult patients with pMAC who were recruited from the prospective, observational cohort registry at Keio University Hospital (UMIN000007546). PMAC was diagnosed on the basis of statements published by the American Thoracic Society (ATS) and Infectious Disease Society of America in 2007 [3]. The Keio University Hospital institutional review board approved the study protocol (# 20110267), and written informed consent was obtained from each patient. All patients with pMAC who participated in 6MWT and completed questionnaires assessing HRQL between May 2012 and November 2013 were included.

### 6MWT

All patients were instructed to walk in a hallway for 6 min according to ATS guidelines [10]. Oxygen saturation by pulse oximetry (SpO_2_) and heart rate (HR) were monitored throughout the 6-min walk. The total distance walked and the oxygen saturation and HR (per minute) measured from start to finish were recorded. The patients were assessed using the Borg Dyspnea Scale at the end of the test [14]. Other parameters such as the distance-saturation product (DSP) [6] and desaturation area (DA) [15] were also calculated.

### HRQL assessments

For the evaluation of HRQL, all patients completed the 36-Item Short Form Health Survey (SF-36), version 2 [16] and SGRQ in Japanese [17]. Both these questionnaires have been validated for use in patients with pMAC disease [5]. The SF-36 comprises physical, mental, and role/social domains, and the respective summary scores were adjusted for Japanese patients [18] before analysis. All scores were transformed to fit a norm-based score of 50 and standard deviation of 10, and lower scores indicated a poorer health status. With regard to SGRQ (range, 0–100), we calculated the total score as well as scores for Symptoms, Activity, and Impacts domains, which evaluate respiratory symptoms, physical activity impairment, and social and psychological disturbances, respectively. Lower scores indicated a better health status.

### Assessment of clinical parameters

Clinical parameters, including sex, age at diagnosis, disease duration, body mass index (BMI), smoking status, underlying pulmonary diseases, comorbidities assessed using the age-adjusted Charlson comorbidity index (CCI) [19], and sputum smear and culture findings for MAC within the previous year were recorded at the time of enrolment. PFT was performed using an electronic spirometer (Chestac-9800 or HI-801; Chest M.I., Tokyo, Japan) when the patient was in a stable condition after study enrolment. MAC isolates were identified as previously described [20]. High-resolution computed tomography (HRCT) images were evaluated for cavitary lesions and the radiological pattern: nodular/bronchiectatic (NB), fibrocavitary (FC), NB + FC, and unclassified [21].

### Statistical analysis

Correlations between two continuous variables were analyzed using Spearman’s correlation coefficients. Comparisons between two groups were conducted using the Wilcoxon rank sum test. To identify 6MWT parameters that predicted HRQL, parameters that showed a significant association with SGRQ and SF-36 scores in univariate analysis were entered into a stepwise forward and backward multiple regression model for multivariate analysis. All *P*-values were two-tailed, and a value of < 0.05 was considered statistically significant. All statistical analyses were conducted using JMP v11.0 (SAS Institute Japan Ltd., Tokyo, Japan).

## Results

### Patient characteristics and SF-36 and SGRQ scores

In total, 103 patients with pMAC were enrolled. Table 1 shows the clinical characteristics of the patients. The median [interquartile range (IQR)] age of patients was 68 (64–75) years. Eighty (78%) patients were women and 92 (89%) patients were never-smokers. Underlying pulmonary diseases were present in 16 (15%) patients. The median PFT values were within the normal range. On HRCT, 31 (30.1%) patients showed cavitary lesions, and the NB pattern was most commonly observed (81 patients, 78.6%). With regard to HRQL, the median scores for the physical and role/social domains in SF-36 were decreased, whereas the median Symptoms, Activity, and total scores in SGRQ were increased.

**Table 1:** Clinical characteristics of patients with pulmonary *Mycobacterium avium* complex disease (*n* = 103)

Variable	
Age, years	68 (64–75)
Sex, Male/Female	23 (22)/80 (78)
Disease duration, years	5.8 (2.3–10.1)
BMI, kg/m^2^	19.2 (17.5–20.4)
Smoking status	
Never/ Former/ Current	92 (89)/11 (11)/0 (0)
Charlson comorbidity index	4 (4–5)
Underlying pulmonary diseases	
Old pulmonary tuberculosis	11 (11)
Bronchial asthma	4 (4)
Lung cancer	1 (1)
Sputum findings for NTM infection within the previous year	
Smear/culture positivity	31 (30)/62 (60)
%FVC, %	94 (80–107)
%FEV_1_, %	87 (73–98)
FEV_1_/FVC < 70%	35 (34)
%FEV_1_ < 80%	39 (38)
Presence of cavitary lesions	31 (30.1)
Radiological pattern	
NB/FC/NB + FC/unclassified	81 (78.6)/3 (2.9)/15 (14.5)/4 (3.9)
SF-36 scores	
PCS	48 (38–54)
MCS	51 (42–56)
RCS	48 (43–54)
SGRQ scores	
Symptoms	31 (15–48)
Activity	24 (6–48)
Impacts	9 (3–29)
Total	19 (9–36)

Data are shown as number (%) of patients or medians (interquartile ranges)

*BMI* body mass index, *FC* fibrocavitary, *FVC* forced volume capacity, *FEV*_*1*_ forced expiratory volume in 1 s, *NB* nodular/bronchiectatic, *MCS* mental component summary, *NTM* nontuberculous mycobacteria, *PCS* physical component summary, *RCS* role/social component summary, *SF-36* 36-Item Short Form Health Survey, *SGRQ* St. George’s Respiratory Questionnaire

### 6MWT parameters

Table 2 shows the findings of 6WMT for the 103 patients. The median (IQR) 6MWD)was 410 (365–450) m. The median (IQR) initial and lowest SpO_2_ values were 96% (96–97%) and 94% (92–95%), respectively, while the median (IQR) initial and final HR values were 76 (67–85) and 106 (97–116) beats/minute, respectively. The median (IQR) final Borg scale (FBS) score, DSP, and DA were 0.5 (0.5–2), 385 (338–423) m%, and 32 (27–40) units.

**Table 2:** Results of the six-minute walk test for patients with pulmonary *Mycobacterium avium* complex disease (*n* = 103)

Variables	
6MWD, m	410 (365–450)
Initial SpO_2_, %	96 (96–97)
Lowest SpO_2_, %	94 (92–95)
Initial heart rate, beats/minute	76 (67–85)
Final heart rate, beats/minute	106 (97–116)
Final Borg scale score	0.5 (0.5–2)
DSP, m%	385 (338–423)
DA, units	32 (27–40)

Data are shown as medians (interquartile ranges)

*6MWD* six-minute walk distance, *DA* desaturation area, *DSP* distance-saturation product, *SpO*_*2*_ oxygen saturation by pulse oximetry

### Correlations among 6MWT parameters and clinical parameters

Table 3 shows Spearman’s correlations among 6MWT parameters and clinical parameters for the 103 patients. 6MWD showed a strong correlation with DSP, DA, and the lowest SpO_2_. The initial SpO_2_ was moderately correlated with the lowest SpO_2_ or DA and the initial and final HRs. Age was significantly correlated with 6MWD, the FBS score, and DSP. Disease duration and age-adjusted CCI were also weakly correlated with the FBS score. Finally, the percentage functional volume capacity (%FVC) and percentage forced expiratory volume in 1 s (%FEV_1_) showed significant but weak correlations with the initial and lowest SpO_2_ values, DSP, and DA.

**Table 3:** Spearman’s correlations among six-minute walk test parameters and clinical parameters for patients with pulmonary *Mycobacterium avium* complex disease (*n* = 103)

	Age	Disease duration	BMI	CCI	%FVC	%FEV_1_	6MWD	Initial SpO_2_	Lowest SpO_2_	Initial HR	Final HR	FBS	DSP	DA
Age														
Disease duration	− 0.09													
BMI	0.01	0.10												
CCI	0.32^‡^	− 0.04	0.09											
%FVC	− 0.06	− 0.08	0.21^*^	− 0.05										
%FEV_1_	0.05	−0.19	0.02	− 0.01	0.78^§^									
6MWD	−0.34^‡^	−0.03	− 0.16	− 0.16	0.18	0.21^*^								
Initial SpO2	−0.03	−0.18	−0.12	−0.05	0.21^*^	0.32^‡^	0.08							
Lowest SpO2	−0.07	−0.11	− 0.05	− 0.08	0.23^*^	0.27^†^	0.03	0.44^§^						
Initial HR	−0.06	0.06	− 0.24^*^	− 0.11	−0.13	−0.05	−0.01	−0.17	−0.17					
Final HR	−0.16	−0.04	−0.07	−0.13	−0.18	−0.11	0.18	−0.17	−0.17	0.56^§^				
FBS	0.28^†^	0.23^*^	− 0.09	0.28^†^	−0.22^*^	− 0.12	−0.17	−0.05	−0.08	−0.002	−0.04			
DSP	−0.35^‡^	−0.05	−0.15	−0.17	0.23^*^	0.26^†^	0.99^§^	0.14	0.16	−0.03	0.16	−0.17		
DA	0.06	0.17	0.07	0.08	−0.25^*^	− 0.29^†^	−0.01	−0.54^§^	−0.95^§^	0.20^*^	0.24^*^	0.11	−0.14	

*6MWD* six-minute walk distance, *6MWT* six-minute walk test, *BMI* body mass index, *BS* Borg scale, *CCI* Charlson comorbidity index, *DA* Desaturation area, *DSP* Distance-saturation product, *FBS* final Borg scale, *FEV*_*1*_ forced expiratory volume in 1 s, *FVC* forced volume capacity, *HR* heart rate, *SpO*_*2*_ oxygen saturation by pulse oximetry

^*^P < 0.05, ^†^*P* < 0.01, ^‡^*P* < 0.001, ^§^*P* < 0.0001

### Correlations among 6MWT parameters or clinical parameters and SF-36 and SGRQ scores

Table 4 shows the correlations among 6MWT parameters or clinical parameters and SF-36 and SGRQ scores for the 103 patients. The physical component summary (PCS) score in SF-36 was strongly correlated with the Activity and total scores in SGRQ. The mental component summary (MCS) and role/social component summary (RCS) scores were also significantly correlated with the SGRQ scores, although the correlations were weaker than that of the PCS score. All SGRQ scores showed a significant negative correlation with 6MWD [ρ = (− 0.54)–(− 0.32)] and DSP [ρ = (− 0.29)–(− 0.55)] and a significant positive correlation with the FBS score (ρ = 0.34–0.58). The PCS and RCS scores in SF-36 showed a significant positive correlation with 6MWD (ρ = 0.39 and ρ = 0.20, respectively) and a significant negative correlation with the FBS score (ρ = − 0.50 and ρ = − 0.24, respectively). The PCS score also showed a significant positive correlation with DSP (ρ = 0.40). Regarding the correlations among clinical parameters and SF-36 and SGRQ scores, all SGRQ scores showed a significant negative correlation with %FVC [ρ = (− 0.43)–(− 0.28)] and %FEV_1_ [ρ = (− 0.35)–(− 0.26)]. The Activity, Impacts, and Total scores in SGRQ showed significant positive correlation (ρ = 0.22–0.45) and the PCS score in SF-36 showed significant negative correlation (ρ = − 0.51) with age. Moreover, the Symptoms, Impacts, and Total scores in SGRQ showed significant negative correlation [ρ = (− 0.22)–(− 0.30)] and the RCS score in SF-36 showed significant positive correlation (ρ = 0.20) with BMI. The Activity score in SGRQ showed significant positive correlation (ρ = 0.33) and the PCS score in SF-36 showed significant negative correlation (ρ = − 0.35) with age-adjusted CCI. We also analyzed the data for the never smoker group (*n* = 92) alone in Additional file 1: Table S4. Correlations among 6MWT parameters or clinical parameters and SF-36 and SGRQ scores were the same as that for all patients including former-smoker patients (*n* = 103).

**Table 4:** Spearman’s correlations among six-minute walk test parameters or clinical parameters and 36-Item Short Form Health Survey and St George’s Respiratory Questionnaire scores for patients with pulmonary *Mycobacterium avium* complex disease (*n* = 103)

		SGRQ				SF-36		
		Symptoms	Activity	Impacts	Total	PCS	MCS	RCS
SGRQ	Symptoms							
	Activity	0.51^§^						
	Impacts	0.68^§^	0.67^§^					
	Total	0.77^§^	0.88^§^	0.91^§^				
SF-36	PCS	−0.44^§^	−0.73^§^	−0.60^§^	−0.70^§^			
	MCS	−0.32^‡^	−0.27^†^	−0.28^†^	−0.29^†^	0.08		
	RCS	−0.28^†^	−0.42^§^	−0.40^§^	−0.44^§^	0.25^*^	0.15	
6WMT	6MWD	−0.27^†^	−0.54^§^	−0.32^†^	−0.44^§^	0.39^§^	0.08	0.20^*^
	Initial SpO_2_	0.0002	−0.12	−0.08	−0.09	0.05	−0.15	−0.09
	Lowest SpO_2_	−0.15	−0.14	−0.21	−0.19	0.10	−0.01	−0.05
	Initial HR	0.16	0.03	0.15	0.11	−0.09	0.03	−0.06
	Final HR	0.11	−0.04	0.04	0.03	0.07	0.00	−0.02
	FBS	0.34^‡^	0.56^§^	0.54^§^	0.58^§^	−0.50^§^	−0.17	−0.24^*^
	DSP	−0.29^†^	−0.55^§^	−0.33^‡^	−0.46^§^	0.40^§^	0.06	0.19
	DA	0.14	0.12	0.19	0.17	−0.09	0.04	0.07
Clinical parameters	Age	0.09	0.45^§^	0.22^*^	0.36^‡^	−0.51^§^	0.10	−0.18
	Disease duration	0.16	0.13	0.11	0.18	−0.06	− 0.02	−0.01
	BMI	−0.30^†^	−0.08	− 0.27^†^	−0.22^*^	0.14	0.07	0.20^*^
	CCI	−0.09	0.33^‡^	0.13	0.19	−0.35^‡^	0.04	−0.05
	%FVC	−0.41^§^	−0.28^†^	− 0.43^§^	−0.43^§^	0.15	−0.03	0.07
	%FEV_1_	−0.34^‡^	−0.26^†^	− 0.32^‡^	−0.35^‡^	0.07	−0.03	− 0.003

*6MWD* six-minute walk distance, *6MWT* six-minute walk test, *BMI* body mass index, *CCI* Charlson comorbidity index, *DA* Desaturation area, *DSP* Distance-saturation product, *FBS* final Borg scale, *FEV*_*1*_ forced expiratory volume in 1 s, *FVC* forced volume capacity, *MCS* mental component summary, *PCS* physical component summary, *RCS* role/social component score, *SF-36* 36-Item Short Form Health Survey, *SGRQ* St. George’s Respiratory Questionnaire, *SpO*_*2*_ oxygen saturation by pulse oximetry

^*^P < 0.05, ^†^*P* < 0.01, ^‡^*P* < 0.001, ^§^*P* < 0.0001

### Comparisons of 6MWT parameters and questionnaire scores between patients with cavitary lesions and those without

We also evaluated whether 6MWT parameters and the questionnaire scores differed between patients with cavitary lesions (*n* = 31, 30.1%) on HRCT and those without (*n* = 72, 69.9%; Additional file 1: Table S6), because cavitary lesions have been reported as a prognostic factor for pMAC disease [22, 23]. While 6MWT parameters except the initial HR showed no significant differences between the two groups, the Symptoms and Impacts scores in SGRQ were significantly higher in patients with cavitary lesions than in those without.

### Multivariate analysis for predictors of SGRQ and SF-36 scores

We performed stepwise multiple regression analysis to determine the association of SGRQ and SF-36 scores with age, sex, age-adjusted CCI, BMI, smoking status, disease duration, underlying pulmonary diseases, positive sputum smear or culture findings, presence of cavitary lesions, pulmonary function, 6MWD, DSP, and the FBS score (Table 5). The MCS and RCS scores were excluded because they only showed weak correlations with 6WMT parameters. We chose 6MWT parameters and clinical parameters for stepwise multiple regression analysis that showed significant correlations with SGRQ or SF-36 scores as shown in Table 4 or that were thought to be clinically important from previous studies. %FVC, sex, and BMI were found to be significant predictors of the Symptoms score in SGRQ, accounting for 21.2% variance, while DSP, the FBS score, and BMI were found to be significant predictors of the Activity score in SGRQ, accounting for 54.0% variance. The FBS score, %FVC, 6MWD, and BMI were found to be significant predictors of the Impacts score in SGRQ, accounting for 38.9% variance. The FBS score, 6MWD, BMI, and %FVC were found to be significant predictors of the SGRQ total score, accounting for 49.4% variance. Finally, the FBS score, 6MWD, age, sex, and disease duration were found to be significant predictors of the physical component summary (PCS) score in SF-36, accounting for 49.2% variance. We also analyzed the data for the never smoker group (*n* = 92) alone in Additional file 1: Table S5. Correlations among 6MWT parameters and SF-36 and SGRQ scores were the same as that of all patients including former-smoker patients (*n* = 103). Hence, it was concluded that 6MWD and the FBS score are useful 6MWT parameters for the prediction of HRQL in patients with pMAC disease in all patients, including the never smoker group (*n* = 92) and former-smoker patients (*n* = 103) (Table 5).

**Table 5:** Multivariate analysis for predictors of 36-Item Short Form Health Survey and St George’s Respiratory Questionnaire scores for patients with pulmonary *Mycobacterium avium* complex disease (*n* = 103)

HRQL	Determinants	*P*-value	Cumulative R^2^, %
SGRQ scores			
Symptoms	%FVC	0.0002	14.6
	Sex	0.0076	21.2
	BMI	0.0303	27.9
Activity	DSP	< 0.0001	33.5
	FBS	< 0.0001	51.3
	BMI	0.0248	54.0
Impacts	FBS	< 0.0001	20.6
	%FVC	0.0020	28.7
	6MWD	0.0192	33.0
	BMI	0.0048	38.9
Total	FBS	< 0.0001	24.3
	6MWD	< 0.0001	39.2
	BMI	0.0005	47.0
	%FVC	0.0483	49.4
SF-36			
PCS	FBS	< 0.0001	22.4
	6MWD	< 0.0001	36.2
	Age	0.0014	43.2
	Sex	0.0179	46.8
	Disease duration	0.0462	49.2

*6MWD* six-minute walk distance, *6MWT* six-minute walk test, *BMI* body mass index, *DA* desaturation area, *DSP* distance-saturation product, *FEV*_*1*_ forced expiratory volume in 1 s, *FBS* final Borg scale, *FVC* forced volume capacity, *PCS* physical component summary, *SGRQ* St. George’s Respiratory Questionnaire, *SF-36* 36-Item Short Form Health Survey, *SpO*_*2*_ oxygen saturation by pulse oximetry

## Discussion

In the present study, we evaluated the relationship of 6MWT parameters with clinical parameters, including PFT findings, and HRQL in patients with pMAC disease. The findings revealed that 6MWD and the FBS score showed strong correlations with the PCS score in SF-36 and all SGRQ scores except for the Symptoms score. Furthermore, %FVC and BMI, which weakly correlated with 6MWD and the FBS score, were important predictors of the SGRQ scores.

The relationship between 6MWD, which is classically used as the primary outcome of 6MWT, and HRQL assessed using different tools (including SGRQ and SF-36) has been determined in patients with various chronic pulmonary diseases [24, 25], and it was found to correlate negatively with the total SGRQ score in patients with COPD and ILD [26–28]. A previous study on pMAC disease reported that 6WMD significantly correlated with the Activity (ρ = − 0.45) and total (ρ = − 0.31) scores in SGRQ [13], while in another study, 6MWD showed a significant positive correlation with the total SF-36 score (ρ = 0.37) in patients with sarcoidosis [29]. Consistent with findings in previous studies, our data demonstrated that 6MWD was significantly correlated with SGRQ scores and the PCS score in SF-36 and was one of the important predictors of HRQL according to multivariate analysis.

In addition to 6WMD, we found that the FBS score was also an important predictor of SGRQ scores and the PCS score in SF-36. Dyspnea reflects the physiology of exercise limitation as well as the impact of exercise limitation on daily life [30]. In addition, scores for dyspnea assessed using the Borg scale exhibit good reliability and are used as a marker of patient-reported fatigue that reflects a local muscle phenomenon as well as general fatigue in patients with chronic pulmonary diseases [14]. Previous studies reported a significant correlation between the FBS score and all SGRQ scores (ρ = 0.33–0.56) in patients with COPD [27] and the total SF-36 score (ρ = 0.45) in patients with sarcoidosis [29].

Our multivariate analysis revealed that both 6MWD and the FBS score were important predictors of HRQL parameters. 6MWD is an objective parameter showing significant correlations with measures of peak work capacity in cardiopulmonary exercise tests [24]. Furthermore, a study on COPD revealed that 6MWD was associated with not only lung function but also lower limb strength, including quadriceps strength and the lean leg mass [31]. On the other hand, the Borg scale is a subjective parameter reflecting subjective breathlessness, perceived exertion, and fatigue [14, 24, 32]. A previous study on severe COPD and asthma indicated that the Borg scale could not be reliably predicted by desaturation and PFT findings [32]. In fact, a study on COPD indicated that both 6MWD and the FBS score were significant independent prognostic factors [33]. Thus, 6MWD reflects objective exercise tolerance better than does the FBS score, and both are useful parameters for the assessment of patients with pMAC disease.

Our multivariate analysis also found that %FVC and BMI, which have been reported as important clinical factors in previous studies, were predictors of SGRQ scores [13, 20, 23], [34, 35]. In a previous study, FVC showed a negative correlation with SGRQ scores [13], consistent with the findings in this study. Moreover, a substantial decline in FEV_1_ and FVC was associated with treatment failure in another study [34]. BMI has been reported as a poor prognostic factor for pulmonary NTM infection [20, 23, 35]. Notably, our results indicated partially significant but weak correlations among BMI, 6WMD or the FBS score, and PFT findings. Therefore, %FVC and BMI, in addition to 6MWD and the FBS score, may be useful parameters for clinical assessments, including HRQL evaluation.

Our study has several potential limitations. First, we may have excluded patients with more severe pMAC disease, considering we only included patients who could complete PFT, 6MWT, and HRQL questionnaires. Second, this was a cross-sectional study; therefore, we could not determine causal associations, particularly with regard to the influence of treatment. Third, our study mainly included female patients, consistent with the participants of previous studies about pMAC disease [1, 35–37]. Comparison between sexes shows significantly lower BMI in women (Additional file 1: Table S7); this may have affected our results, because BMI is a significant predictor of all SGRQ scores (Table 5). Moreover, we could not evaluate the impact of 6MWT parameters on the prognosis. However, a shorter 6MWD is reported to be strongly correlated with an increased risk of mortality in patients with other chronic pulmonary diseases, including COPD, ILD, and pulmonary hypertension [24, 25, 38–40]. Therefore, we believe that 6MWT may be useful for the assessment of prognosis in patients with pMAC disease.

## Conclusions

Our findings suggest that 6MWD and the FBS score are useful 6MWT parameters for the prediction of HRQL in patients with pMAC disease. However, further studies are needed to investigate the impact of 6WMT parameters on disease progression, treatment responses, and prognosis.

## Additional file

Additional file 1: Supplemental analyses. **Table S1.** Clinical characteristics of patients with pulmonary *Mycobacterium avium* complex disease in the never smoker group (*n* = 92). **Table S2.** Results of the six-minute walk test for patients with pulmonary *Mycobacterium avium* complex disease in the never smoker group (*n* = 92). **Table S3.** Spearman’s correlations among six-minute walk test parameters and clinical parameters for patients with pulmonary *Mycobacterium avium* complex disease in the never smoker group (*n* = 92). **Table S4.** Spearman’s correlations among six-minute walk test parameters and 36-Item Short Form Health Survey and St George’s Respiratory Questionnaire scores for patients with pulmonary *Mycobacterium avium* complex disease in the never smoker group (*n* = 92). **Table S5.** Multivariate analysis for predictors of 36-Item Short Form Health Survey and St George’s Respiratory Questionnaire scores for patients with pulmonary *Mycobacterium avium* complex disease in the never smoker group (*n* = 92). **Table S6.** Comparisons of six-minute walk test parameters and 36-Item Short Form Health Survey and St George’s Respiratory Questionnaire scores with or without cavitary lesions (*n* = 103). **Table S7.** Comparisons of clinical characteristics in pulmonary *Mycobacterium avium* complex disease patients between male and female (*n* = 103). **Table S8.** Multivariate analysis for predictors of 36-Item Short Form Health Survey and St George’s Respiratory Questionnaire scores for patients with pulmonary *Mycobacterium avium* complex disease in female alone (*n* = 80). **Table S9.** Comparisons of 36-Item Short Form Health Survey and St George’s Respiratory Questionnaire scores with or without several clinical parameters (*n* = 103). (DOCX 55 kb)

## Abbreviations

6MWD: six-minute walk distance
6MWT: six-minute walk test
ATS: American Thoracic Society
BMI: body mass index
CCI: Charlson comorbidity index
COPD: chronic obstructive pulmonary disease
DA: desaturation area
DSP: distance-saturation product
FC: fibrocavitary
FEV_1_: forced expiratory volume in 1 s
FVC: functional volume capacity
HRQL: health-related quality of life
ILD: interstitial lung disease
IQR: interquartile range
MAC: *Mycobacterium avium* complex
MCS: mental component summary
NB: nodular/bronchiectatic
PCS: physical component summary
PFT: pulmonary function test
RCS: role/social component summary
SF-36: 36-Item Short Form Health Survey
SGRQ: St. George’s Respiratory Questionnaire
SpO_2_: oxygen saturation by pulse oximetry
