# Supplementary material for: Association between six-minute walk test parameters and the health-related quality of life in patients with pulmonary Mycobacterium avium complex disease
Source: BMC Pulm Med. 2018 Jul 13;18:114. doi: 10.1186/s12890-018-0686-5 (PMC6045883; doi:10.1186/s12890-018-0686-5)
Supplement: Supplementary file 1 — Supplemental analyses. Table S1. Clinical characteristics of patients with pulmonary Mycobacterium avium complex disease in the never smoker group (n = 92). Table S2. Results of the six-minute walk test for patients with pulmonary Mycobacterium avium complex disease in the never smoker group (n = 92). Table S3. Spearman’s correlations among six-minute walk test parameters and clinical parameters for patients with pulmonary Mycobacterium avium complex disease in the never smoker group (n = 92). Table S4. Spearman’s correlations among six-minute walk test parameters and 36-Item Short Form Health Survey and St George’s Respiratory Questionnaire scores for patients with pulmonary Mycobacterium avium complex disease in the never smoker group (n = 92). Table S5. Multivariate analysis for predictors of 36-Item Short Form Health Survey and St George’s Respiratory Questionnaire scores for patients with pulmonary Mycobacterium avium complex disease in the never smoker group (n = 92). Table S6. Comparisons of six-minute walk test parameters and 36-Item Short Form Health Survey and St George’s Respiratory Questionnaire scores with or without cavitary lesions (n = 103). Table S7. Comparisons of clinical characteristics in pulmonary Mycobacterium avium complex disease patients between male and female (n = 103). Table S8. Multivariate analysis for predictors of 36-Item Short Form Health Survey and St George’s Respiratory Questionnaire scores for patients with pulmonary Mycobacterium avium complex disease in female alone (n = 80). Table S9. Comparisons of 36-Item Short Form Health Survey and St George’s Respiratory Questionnaire scores with or without several clinical parameters (n = 103). (DOCX 55 kb) [file 12890_2018_686_MOESM1_ESM.docx]

| **Table S1** Clinical characteristics of patients with pulmonary *Mycobacterium avium* complex disease in the never smoker group (n = 92) | |
| --- | --- |
| **Variable** |  |
| Age, years | 68 (64-75) |
| Sex, Male/Female | 15 (16)/77 (84) |
| Disease duration, years | 5.7 (2.3-10.4) |
| BMI, kg/m^2^ | 19.2 (17.4-20.4) |
| Charlson comorbidity index | 4 (4-5) |
| Underlying pulmonary diseases |  |
| Old pulmonary tuberculosis | 6 (93) |
| Bronchial asthma | 2 (98) |
| Lung cancer | 1 (99) |
| Sputum findings for NTM infection within the previous year |  |
| Smear/culture positivity | 29 (35)/56 (67) |
| %FVC, % | 94 (80-107) |
| %FEV_1_, % | 87 (73-98) |
| FEV_1_/FVC < 70% | 31 (34) |
| %FEV_1_ < 80% | 35 (38) |
| Presence of cavitary lesions | 30 (33) |
| Radiological pattern |  |
| NB/FC/NB+FC/unclassified | 72 (78)/ 3(3)/ 15(16)/ 2(2) |
| SF-36 scores |  |
| PCS | 49 (38-54) |
| MCS | 48 (43-55) |
| RCS | 51 (42-56) |
| SGRQ scores |  |
| Symptoms | 31 (16-48) |
| Activity | 24 (6-48) |
| Impacts | 9 (3-29) |
| Total | 19 (9-36) |
| Data are shown as number (%) of patients or medians (interquartile ranges).  BMI, body mass index; FC, fibrocavitary; FVC, forced volume capacity; FEV_1_, forced expiratory volume in 1 s; NB, nodular/bronchiectatic; MCS, mental component summary; NTM, nontuberculous mycobacteria; PCS, physical component summary; RCS, role/social component summary; SF-36, 36-Item Short Form Health Survey; SGRQ, St. George’s Respiratory Questionnaire | |

| **Table S2** Results of the six-minute walk test for patients with pulmonary *Mycobacterium avium* complex disease in the never smoker group (n = 92) | |
| --- | --- |
| **Variables** | |
| 6MWD, m | 410 (366-450) |
| Initial SpO_2_, % | 96 (95-97) |
| Lowest SpO_2_, % | 94 (92-95) |
| Initial heart rate, beats/minute | 78 (67-86) |
| Final heart rate, beats/minute | 107 (97-116) |
| Final Borg scale score | 0.5 (0.5-2) |
| DSP, m% | 385 (339-423) |
| DA, units | 32 (27-39) |
| Data are shown as medians (interquartile ranges).  6MWD, six-minute walk distance; DA, desaturation area; DSP, distance-saturation product; SpO_2_, oxygen saturation by pulse oximetry | |

| **Table S3** Spearman’s correlations among six-minute walk test parameters and clinical parameters for patients with pulmonary *Mycobacterium avium* complex disease in the never smoker group (n = 92) | | | | | | | | | | | | | | |
| --- | --- | --- | --- | --- | --- | --- | --- | --- | --- | --- | --- | --- | --- | --- |
|  | Age | Disease duration | BMI | CCI | %FVC | %FEV_1_ | 6MWD | Initial SpO_2_ | Lowest SpO_2_ | Initial HR | Final  HR | FBS | DSP | DA |
| Age |  |  |  |  |  |  |  |  |  |  |  |  |  |  |
| Disease duration | −0.08 |  |  |  |  |  |  |  |  |  |  |  |  |  |
| BMI | -0.05 | 0.08 |  |  |  |  |  |  |  |  |  |  |  |  |
| CCI | 0.26^*^ | −0.03 | 0.04 |  |  |  |  |  |  |  |  |  |  |  |
| %FVC | −0.06 | −0.06 | 0.19 | −0.06 |  |  |  |  |  |  |  |  |  |  |
| %FEV_1_ | 0.01 | −0.16 | -0.01 | −0.06 | 0.81^§^ |  |  |  |  |  |  |  |  |  |
| 6MWD | −0.38^‡^ | −0.01 | −0.14 | −0.12 | 0.22^*^ | 0.21^*^ |  |  |  |  |  |  |  |  |
| Initial SpO2 | −0.04 | −0.14 | −0.13 | −0.03 | 0.25^*^ | 0.34^‡^ | 0.04 |  |  |  |  |  |  |  |
| Lowest SpO2 | -0.07 | −0.09 | −0.03 | −0.09 | 0.20 | 0.25^*^ | 0.06 | 0.50^§^ |  |  |  |  |  |  |
| Initial HR | −0.01 | 0.01 | −0.23^*^ | −0.07 | −0.15 | −0.01 | −0.01 | −0.15 | −0.20 |  |  |  |  |  |
| Final HR | −0.14 | −0.07 | −0.02 | −0.08 | −0.18 | −0.06 | 0.18 | −0.15 | −0.21^*^ | 0.56^§^ |  |  |  |  |
| FBS | 0.25^*^ | 0.26^*^ | −0.13 | 0.27^†^ | -0.24^*^ | −0.11 | −0.15 | −0.03 | −0.06 | −0.003 | −0.02 |  |  |  |
| DSP | −0.38^‡^ | −0.02 | −0.12 | −0.13 | 0.27^*^ | 0.28^†^ | 0.99^§^ | 0.11 | 0.20 | −0.03 | 0.15 | −0.15 |  |  |
| DA | 0.5 | 0.16 | 0.05 | 0.07 | −0.22^*^ | −0.29^†^ | −0.04 | −0.60^§^ | −0.95^§^ | 0.25^*^ | 0.29^†^ | 0.09 | −0.17 |  |
| ^*^*P* < 0.05, ^†^*P* < 0.01, ^‡^*P* < 0.001, ^§^*P* < 0.0001  6MWD, six-minute walk distance; 6MWT, six-minute walk test; BMI, body mass index; CCI, Charlson comorbidity index; DA, Desaturation area; DSP, Distance-saturation product; FBS, final Borg scale; FEV_1_, forced expiratory volume in 1s; FVC, forced volume capacity; HR, heart rate; SpO_2_, oxygen saturation by pulse oximetry | | | | | | | | | | | | | | |

| **Table S4** Spearman’s correlations among six-minute walk test parameters and 36-Item Short Form Health Survey and St George’s Respiratory Questionnaire scores for patients with pulmonary *Mycobacterium avium* complex disease in the never smoker group (n = 92) | | | | | | | | | |
| --- | --- | --- | --- | --- | --- | --- | --- | --- | --- |
|  |  | SGRQ | | | |  | SF-36 | | |
|  |  | Symptoms | Activity | Impacts | Total |  | PCS | MCS | RCS |
| SGRQ | Symptoms |  |  |  |  |  |  |  |  |
|  | Activity | 0.51^§^ |  |  |  |  |  |  |  |
|  | Impacts | 0.70^§^ | 0.66^§^ |  |  |  |  |  |  |
|  | Total | 0.78^§^ | 0.88^§^ | 0.90^§^ |  |  |  |  |  |
|  |  |  |  |  |  |  |  |  |  |
| SF-36 | PCS | −0.44^§^ | −0.71^§^ | −0.59^§^ | −0.68^§^ |  |  |  |  |
|  | MCS | −0.27^†^ | −0.22^†^ | −0.26^†^ | −0.25^†^ |  | 0.03 |  |  |
|  | RCS | −0.34^‡^ | −0.45^§^ | −0.40^§^ | −0.47^§^ |  | 0.23^*^ | 0.20 |  |
|  |  |  |  |  |  |  |  |  |  |
| 6WMT | 6MWD | −0.24^*^ | −0.51^§^ | −0.33^†^ | −0.43^§^ |  | 0.38^§^ | -0.006 | 0.28^†^ |
|  | Initial SpO_2_ | 0.007 | −0.09 | −0.05 | −0.06 |  | 0.07 | −0.21^*^ | −0.09 |
|  | Lowest SpO_2_ | −0.17 | −0.15 | −0.19 | −0.19 |  | 0.12 | 0.01 | −0.05 |
|  | Initial HR | 0.13 | 0.001 | 0.13 | 0.09 |  | −0.09 | 0.00 | −0.07 |
|  | Final HR | 0.09 | −0.03 | 0.03 | 0.03 |  | 0.03 | 0.03 | −0.02 |
|  | FBS | 0.35^‡^ | 0.56^§^ | 0.55^§^ | 0.59^§^ |  | −0.51^§^ | −0.17 | −0.25^*^ |
|  | DSP | −0.27^*^ | −0.53^§^ | −0.34^‡^ | −0.45^§^ |  | 0.38^‡^ | -0.02 | 0.27^*^ |
|  | DA | 0.18 | 0.14 | 0.17 | 0.18 |  | −0.11 | 0.01 | 0.07 |
| Clinical parameters | Age | 0.14 | 0.50^§^ | 0.25^*^ | 0.40^§^ |  | -0.53^§^ | 0.09 | -0.18 |
|  | Disease duration | 0.13 | 0.12 | 0.09 | 0.16 |  | -0.06 | 0.05 | -0.06 |
|  | BMI | -0.32^†^ | -0.09 | -0.27^*^ | -0.24^*^ |  | 0.17 | 0.12 | 0.22^*^ |
|  | CCI | -0.07 | 0.34^‡^ | 0.15 | 0.21^*^ |  | -0.34^‡^ | 0.04 | -0.05 |
|  | %FVC | -0.41^§^ | -0.31^†^ | -0.43^§^ | -0.44^§^ |  | 0.17 | -0.04 | 0.14 |
|  | %FEV_1_ | -0.29^†^ | -0.25^*^ | -0.29^†^ | -0.34^†^ |  | 0.07 | -0.10 | 0.03 |
| ^*^*P* < 0.05, ^†^*P* < 0.01, ^‡^*P* < 0.001, ^§^*P* < 0.0001  6MWD, six-minute walk distance; 6MWT, six-minute walk test; BMI, body mass index; CCI, Charlson comorbidity index; DA, Desaturation area; DSP, Distance-saturation product; FBS, final Borg scale; FEV_1_, forced expiratory volume in 1s; FVC, forced volume capacity; MCS, mental component summary; PCS, physical component summary; RCS, role/social component score; SF-36, 36-Item Short Form Health Survey; SGRQ, St. George’s Respiratory Questionnaire; SpO_2_, oxygen saturation by pulse oximetry | | | | | | | | | |

| **Table S5** Multivariate analysis for predictors of 36-Item Short Form Health Survey and St George’s Respiratory Questionnaire scores for patients with pulmonary *Mycobacterium avium* complex disease in the never smoker group (n = 92) | | | |
| --- | --- | --- | --- |
| HRQL | Determinants | *P*-value | Cumulative R^2^, % |
| SGRQ scores |  |  |  |
| Symptoms | %FVC | 0.0005 | 14.1 |
|  | BMI | 0.0148 | 20.5 |
|  | 6MWD | 0.0285 | 25.3 |
| Activity | DSP | <0.0001 | 32.2 |
|  | FBS | <0.0001 | 51.2 |
|  | Age | 0.0262 | 54.3 |
| Impacts | FBS | <0.0001 | 21.6 |
|  | %FVC | 0.0035 | 29.8 |
|  | 6MWD | 0.0185 | 34.7 |
|  | BMI | 0.0127 | 39.8 |
| Total | FBS | <0.0001 | 25.0 |
|  | 6MWD | <0.0001 | 40.8 |
|  | BMI | 0.0021 | 47.7 |
| SF-36 scores |  |  |  |
| PCS | Age | <0.0001 | 24.2 |
|  | FBS | 0.0001 | 38.5 |
|  | 6MWD | 0.0036 | 45.3 |
|  | Sex | 0.0287 | 48.6 |
|  | Disease duration | 0.0348 | 51.6 |
| 6MWD, six-minute walk distance; 6MWT, six-minute walk test; BMI, body mass index; DA, desaturation area; DSP, distance-saturation product; FEV_1_, forced expiratory volume in 1s; FBS, final Borg scale; FVC, forced volume capacity; PCS, physical component summary; SGRQ, St. George’s Respiratory Questionnaire; SF-36, 36-Item Short Form Health Survey; SpO_2_, oxygen saturation by pulse oximetry | | | |

| **Table S6** Comparisons of six-minute walk test parameters and 36-Item Short Form Health Survey and St George’s Respiratory Questionnaire scores with or without cavitary lesions (n = 103) | | | |
| --- | --- | --- | --- |
| **Variables** | Cavity (+) (n=31) | Cavity (-) (n=72) | *P*-value |
| 6MWT parameters |  |  |  |
| 6MWD, m | 405 (370-450) | 412 (360-454) | 0.7901 |
| Initial SpO_2_, % | 96 (95-97) | 96 (96-97) | 0.9852 |
| Lowest SpO_2_, % | 93.0 (91-95) | 94 (92-95) | 0.1369 |
| Initial heart rate, beats/minute | 79 (76-86) | 74 (65-85) | 0.0058^†^ |
| Final heart rate, beats/minute | 109 (101-115) | 105 (96-116) | 0.2633 |
| Final Borg scale | 0.5 (0.5-2) | 0.5 (0.5-3) | 0.9641 |
| DSP, m% | 384 (343-408) | 386 (338-427) | 0.6072 |
| DA, units | 32 (28-49) | 32 (26-39) | 0.2540 |
| SF-36 scores |  |  |  |
| PCS | 49 (40-53) | 48 (36-54) | 0.5749 |
| MCS | 45 (41-52) | 49 (44-55) | 0.0775 |
| RCS | 50 (44-55) | 51 (41-56) | 0.7354 |
| SGRQ scores |  |  |  |
| Symptoms | 37 (20-55) | 28 (14-45) | 0.0496^*^ |
| Activity | 29 (17-48) | 21 (6-48) | 0.3132 |
| Impacts | 15 (6-37) | 7 (0-27) | 0.0179^*^ |
| Total | 30 (11-38) | 15.7 (7-34) | 0.0616 |
| Data are shown as number (%) of patients or medians (interquartile ranges).  *P* value for Wilcoxon rank sum test are shown. ^*^*P* < 0.05, ^†^*P* < 0.01.  6MWD, six-minute walk distance; 6MWT, six-minute walk test; DA, desaturation area; DSP, distance-saturation product; HRQL, health-related quality of life; MCS, mental component summary; PCS, physical component summary; RCS, role/social component summary; SF-36, 36-item short form health survey; SGRQ, St. George's Respiratory Questionnaire; SpO_2_, oxygen saturation by pulse oximetry. | | | |

| **Table S7** Comparisons of clinical characteristics in pulmonary *Mycobacterium avium* complex disease patients between male and female (n = 103) | | | |
| --- | --- | --- | --- |
| **Variable** | Male (n=23) | Female (n=80) | *P*-value |
| Clinical parameters |  |  |  |
| Age, years | 76 (68-81) | 66 (63-74) | 0.0017^†^ |
| Disease duration, years | 3.2 (1.5-6.3) | 6.3 (2.8-10.5) | 0.0171^*^ |
| BMI, kg/m^2^ | 19.9 (18.1-22.0) | 18.7 (17.3-20.4) | 0.0303^*^ |
| Charlson comorbidity index | 5 (4-5) | 4 (4-4) | 0.0086^†^ |
| %FVC, % | 94 (77-105) | 94 (81-108) | 0.6151 |
| %FEV_1_, % | 84 (74-92) | 87 (69-100) | 0.4736 |
| 6MWT parameters |  |  |  |
| 6MWD, m | 413 (350-515) | 410 (366-444) | 0.4144 |
| Initial SpO_2_, % | 96 (95-97) | 96 (96-97) | 0.7714 |
| Lowest SpO2, % | 94 (91-94) | 93 (92-95) | 0.3991 |
| Initial heart rate, beats/minute | 76 (62-79) | 78 (67-86) | 0.0752 |
| Final heart rate, beats/minute | 102 (95-115) | 107 (97-116) | 0.2194 |
| Final Borg scale | 0.5 (0.5-3) | 0.5 (0.5-2) | 0.7073 |
| DSP, m% | 388 (322-480) | 385 (339-409) | 0.4843 |
| DA, units | 35 (27-44) | 32 (27-40) | 0.3296 |
| SF-36 scores |  |  |  |
| PCS | 51 (44-55) | 48 (37-53) | 0.2318 |
| MCS | 49 (43-58) | 48 (43-53) | 0.2492 |
| RCS | 52 (42-55) | 51 (40-56) | 0.7394 |
| SGRQ scores |  |  |  |
| Symptoms | 25 (12-35) | 35 (19-51) | 0.0558 |
| Activity | 18 (6-47) | 27 (6-48) | 0.6005 |
| Impacts | 9 (3-20) | 10 (2-34) | 0.3480 |
| Total | 19 (8-25) | 20 (9-38) | 0.3440 |
| Data are shown as number (%) of patients or medians (interquartile ranges).  *P* value for Wilcoxon rank sum test are shown. ^*^*P* < 0.05, ^†^*P* < 0.01.  6MWD, six-minute walk distance; 6MWT, six-minute walk test; BMI, body mass index; DA, desaturation area; DSP, distance-saturation product; FVC, forced volume capacity; FEV_1_, forced expiratory volume in 1 s; MCS, mental component summary; PCS, physical component summary; RCS, role/social component summary; SF-36, 36-Item Short Form Health Survey; SGRQ, St. George’s Respiratory Questionnaire; SpO_2_, oxygen saturation by pulse oximetry. | | | |

| **Table S8** Multivariate analysis for predictors of 36-Item Short Form Health Survey and St George’s Respiratory Questionnaire scores for patients with pulmonary *Mycobacterium avium* complex disease in female alone (n = 80) | | | |
| --- | --- | --- | --- |
| HRQL | Determinants | *P*-value | Cumulative R^2^, % |
| SGRQ scores |  |  |  |
| Symptoms | %FVC | 0.0004 | 17.1 |
|  | DSP | 0.0241 | 23.3 |
| Activity | DSP | <0.0001 | 31.7 |
|  | FBS | <0.0001 | 53.3 |
|  | Age | 0.0059 | 58.5 |
|  | BMI | 0.0356 | 61.3 |
| Impacts | FBS | 0.0002 | 18.7 |
|  | DSP | 0.0003 | 33.3 |
|  | BMI | 0.0085 | 40.0 |
| Total | FBS | <0.0001 | 26.7 |
|  | 6MWD | <0.0001 | 43.8 |
|  | BMI | 0.0021 | 50.7 |
|  | Age | 0.0329 | 54.1 |
| SF-36 scores |  |  |  |
| PCS | Age | <0.0001 | 34.7 |
|  | FBS | <0.0001 | 51.1 |
|  | 6MWD | 0.0045 | 54.1 |
| 6MWD, six-minute walk distance; 6MWT, six-minute walk test; BMI, body mass index; DA, desaturation area; DSP, distance-saturation product; FEV_1_, forced expiratory volume in 1s; FBS, final Borg scale; FVC, forced volume capacity; PCS, physical component summary; SGRQ, St. George’s Respiratory Questionnaire; SF-36, 36-Item Short Form Health Survey; SpO_2_, oxygen saturation by pulse oximetry | | | |

| **Table S9** Comparisons of 36-Item Short Form Health Survey and St George’s Respiratory Questionnaire scores with or without several clinical parameters (n = 103) | | | | | | | | | | | | | | | |
| --- | --- | --- | --- | --- | --- | --- | --- | --- | --- | --- | --- | --- | --- | --- | --- |
| **Variables** | Cavity | | *P*-value | Sputum smear | | *P*-value | Sputum culture | | *P*-value | Smoking history | | *P*-value | UPD | | *P*-value |
|  | (+)  (n=31) | (-)  (n=72) |  | (+)  (n= 31) | (-)  (n=72) |  | (+)  (n=62) | (-)  (n=41) |  | (+)  (n=11) | (-)  (n=92) |  | (+)  (n=13) | (-)  (n=90) |  |
| SF-36 scores |  |  |  |  |  |  |  |  |  |  |  |  |  |  |  |
| PCS | 49  (40-53) | 48  (36-54) | 0.5749 | 46  (37-55) | 47  (38-52) | 0.8489 | 47  (36-53) | 47  (39-52) | 0.8689 | 44  (37-52) | 49  (38-54) | 0.4944 | 37  (30-48) | 49  (40-54) | 0.0332^*^ |
| MCS | 45  (41-52) | 49  (44-55) | 0.0775 | 46  (40-53) | 50  (44-54) | 0.0971 | 47  (41-53) | 49  (45-57) | 0.0806 | 48  (46-57) | 48  (43-53) | 0.6423 | 49  (43-56) | 48  (43-54) | 0.8737 |
| RCS | 50  (44-55) | 51  (41-56) | 0.7354 | 51  (38-55) | 49  (40-56) | 0.8489 | 49  (39-56) | 51  (43-56) | 0.5656 | 47  (37-55) | 51  (42-56) | 0.5182 | 40  (35-49) | 52  (44-56) | 0.0059^†^ |
| SGRQ scores |  |  |  |  |  |  |  |  |  |  |  |  |  |  |  |
| Symptoms | 37  (20-55) | 28  (14-45) | 0.0496^*^ | 37  (21-54) | 32  (16-47) | 0.3634 | 32  (20-46) | 35  (16-56) | 0.5446 | 28  (15-51) | 31  (15-48) | 0.7853 | 32  (13-39) | 30  (16-51) | 04684 |
| Activity | 29  (17-48) | 21  (6-48) | 0.3132 | 29  (6-48) | 27  (6-48) | 0.9366 | 29  (10-48) | 24  (6-48) | 0.4291 | 26  (13-47) | 24  (6-48) | 0.8347 | 42  (30-48) | 21  (6-47) | 0.0189^*^ |
| Impacts | 15  (6-37) | 7  (0-27) | 0.0179^*^ | 13  (0-38) | 10  (4-29) | 0.7896 | 10  (4-30) | 12  (3-33) | 0.6949 | 20  (2-33) | 9  (3-29) | 0.7436 | 24  (3-29) | 10  (3-30) | 0.8263 |
| Total | 30  (11-38) | 15.7  (7-34) | 0.0616 | 19  (10-36) | 21  (10-44) | 0.7091 | 20  (10-36) | 23  (8-39) | 0.9751 | 24  (7-38) | 19  (9-36) | 0.8434 | 24  (15-39) | 18  (8-36) | 0.2555 |
| Data are shown as number (%) of patients or medians (interquartile ranges).  *P* value for Wilcoxon rank sum test are shown. ^*^*P* < 0.05, ^†^*P* < 0.01.  6MWD, six-minute walk distance; 6MWT, six-minute walk test; DA, desaturation area; DSP, distance-saturation product; HRQL, health-related quality of life; MCS, mental component summary; PCS, physical component summary; RCS, role/social component summary; SF-36, 36-item short form health survey; SGRQ, St. George's Respiratory Questionnaire; SpO_2_, oxygen saturation by pulse oximetry; UPD, underlying pulmonary disease. | | | | | | | | | | | | | | | |
